# The frail older person does not exist: development of frailty profiles with latent class analysis

**DOI:** 10.1186/s12877-018-0776-5

**Published:** 2018-04-04

**Authors:** W. M. Looman, I. N. Fabbricotti, J. W. Blom, A. P. D. Jansen, J. E. Lutomski, S. F. Metzelthin, R. Huijsman, on behalf of the TOPICS-MDS Research Consortium

**Affiliations:** 1 Erasmus School of Health Policy & Management, Erasmus University Rotterdam, PO Box 2040, 3000 CA Rotterdam, The Netherlands; 2 Department of Public Health and Primary Care, Leiden University Medical Center, PO Box 9600, 2300 RC Leiden, The Netherlands; 3 Department of General Practice and Elderly Care Medicine, Amsterdam Public Health research Institute, VU University Medical Center, PO Box 7057, 1007 MB Amsterdam, The Netherlands; 4 Radboud Biobank, Radboud University Medical Center, Geert Grooteplein 10, 6525 GA Nijmegen, The Netherlands; 5 Department of Health Services Research, Care and Public Health Research Institute (CAPHRI), Maastricht University, PO Box 616, 6200 MD Maastricht, The Netherlands

**Keywords:** Frailty, Older people, Profiles, Latent class analysis

## Abstract

**Background:**

A fundamental issue in elderly care is targeting those older people at risk and in need of care interventions. Frailty is widely used to capture variations in health risks but there is no general consensus on the conceptualization of frailty. Indeed, there is considerable heterogeneity in the group of older people characterized as frail. This research identifies frailty profiles based on the physical, psychological, social and cognitive domains of functioning and the severity of the problems within these domains.

**Methods:**

This research was a secondary data-analysis of older persons derived from The Older Person and Informal Caregiver Minimum Dataset. Selected respondents were 60 years and older (*n* = 43,704; 59.6% female). The following variables were included: self-reported health, cognitive functioning, social functioning, mental health, morbidity status, and functional limitations. Using latent class analysis, the population was divided in subpopulations that were subsequently discussed in a focus group with older people for further validation.

**Results:**

We distinguished six frailty profiles: *relatively healthy*; *mild physically frail*; *psychologically frail*; *severe physically frail*; *medically frail* and *multi-frail*. The *relatively healthy* had limited problems across all domains. In three profiles older people mostly had singular problems in either the physical or psychological domain and the severity of the problems differed. Two remaining profiles were multidimensional with a combination of problems that extended to the social and cognitive domains.

**Conclusions:**

Our research provides an empirical base for meaningful frailty profiles. The profiles showed specific patterns underlying the problems in different domains of functioning. The heterogeneous population of frail older people has differing needs and faces different health issues that should be considered to tailor care interventions. Evaluation research of these interventions should acknowledge the heterogeneity of frailty by profiling.

**Electronic supplementary material:**

The online version of this article (10.1186/s12877-018-0776-5) contains supplementary material, which is available to authorized users.

## Background

Population ageing and care for older people pose major challenges for health care systems globally. The number of older persons is increasing rapidly; the number of people aged 60 years or over will increase by 56% between 2015 and 2030 and the population over 80 years of age (oldest–old) will increase even faster [1]. There is wide variety within this increasing population; older people experience their health considerably differently [2] and their needs differ as well [3]. Consequently, a fundamental issue in elderly care is targeting those older people at risk and in need of care interventions. The question remains: which intervention works best for whom? Traditionally, chronological age was used as a marker for targeted care. However, age is not specific enough because the ageing process varies substantially between individuals [4].

Consequently, the notion of frailty was introduced to better target older people in need of care interventions [5, 6], because frailty better captures variations in health risks than chronological age [7]. Frailty is a complex condition involving the interaction of multiple problems in different domains of functioning [7]. Frail people are at risk for adverse outcomes such as falls, functional decline, hospitalization, institutionalization and mortality [8–10]. Yet, despite agreeing on the complexity of frailty and its relation to adverse outcomes, health care professionals, policy makers and researchers have not achieved consensus on the conceptualization of frailty [11]. Frailty has become a buzzword [12] and considerable heterogeneity exists within the group of older people labelled frail.

To elucidate the heterogeneity within the frail population, researchers have explored the physical, psychological and social domains of frailty. Frailty has been related to the physical domain of functioning with characteristics such as unintentional weight loss or exhaustion [8]. Other researchers have conceptualized frailty from a broader perspective which also includes the psycho-social domains [5, 9, 13]. Important in the daily functioning of older people, these domains are characterized by memory loss, and feelings of anxiety or loneliness. Still, the distinction between the separate domains does not demonstrate the full complexity of frailty. The domains might influence or reinforce each other and thus it remains unclear which specific combinations lead to adverse outcomes [9, 14]. Frailty has been conceptualized as an accumulation of deficits in these domains and a frailty index can be calculated by dividing the number of deficits a person has by the maximum number of deficits [15, 16]. Also, to identify older people in need of interventions, frailty measurement instruments are used that sum the number of health problems and do not differentiate between the underlying problems [17].

Further specification of frailty by defining *profiles* of frail older people contributes to the ongoing debate on the conceptualization of frailty and could improve interventions. To date, the heterogeneity in the frail population is not fully acknowledged in care interventions and populations substantially differ between and within interventions [18]. Profiling, or distinguishing subpopulations, is common in other disciplines such as social sciences, economics and medical sciences [14]. Recently, subpopulations have also been used in studies of the older population. However, this research focused specifically on chronic conditions [19–21], general health status [14, 22] and physical frailty [23]. These studies did not include the psychological and social domains [14, 19, 22] whereas researchers have emphasized that frailty also involves both these domains of functioning [5, 9, 13].

Therefore, the aim of this study is to identify frailty profiles, constructed on the basis of not only functional limitations, multi-morbidity and self-reported health, but also mental, cognitive and social functioning. Our research expands current knowledge in creating a frailty taxonomy which includes the full range of domains of functioning and the severity of the problems within these domains. These identified profiles could be applied in tailoring interventions such as integrated care interventions and should form part of the evaluation of these interventions.

## Methods

### Data source

For this study we performed a secondary data-analysis on The Older Person and Informal Caregiver Survey Minimum Dataset (TOPICS-MDS), a large data-sharing initiative in the Netherlands (for more information see [24]). In 2008, the Dutch Ministry of Health, Welfare and Sports started the National Care for the Elderly Programme (NCEP) which aimed at reorganizing health and social care according to the needs of older people. Between 2008 and 2014 several implementation and research projects were carried out and funded by the NCEP. Within the NCEP, the TOPICS-MDS instrument was developed, a standardized instrument to study the effects of these projects on older people and their informal caregivers. The instrument was based on other validated instruments on morbidity, quality of life, functional limitations, mental health, social functioning and health service utilization. Researchers in all projects collected the data consistent with the TOPICS-MDS so a national, uniform dataset was created. The TOPICS-MDS currently contains pooled data from 54 research projects which differ across study design, sampling framework an inclusion criteria. TOPICS-MDS is a fully anonymized dataset available for public access, and therefore the analysis in this study is exempt from ethical review (Radboud University Medical Centre Ethical Committee review reference number: CMO: 2012/120) [24]. For our study, we selected the baseline data of the respondents aged 60 years and older (*n* = 43,704).

### Measurements

Baseline measurements entailed: *Self-reported health* is assessed with two items from RAND-36. The first item allows older people to evaluate their own current general health in the following answer categories: excellent; very good; good; fair; poor. The second item is self-reported health compared to 1 year ago with five answer categories: much better; somewhat better; about the same; somewhat worse; much worse [25]. *Cognitive functioning* is measured by one item from EQ-5D + c focused on problems with memory, attention and thinking, and had three answer categories: no problems; some problems; extreme problems with memory, attention and thinking [26]. *Social functioning* is measured with one item on how often social activities are hampered by physical health or emotional problems. The possible answers are: none of the time, a little of the time, some of the time, most of the time, all of the time [25]. *Mental health* is measured on a five-item RAND-36 scale with items that question how often the respondents have felt nervous, calm and peaceful, down-hearted and blue, happy, or so down in the dumps that nothing could cheer them up. The scores range from 0 to 100 and a higher score implies better mental health [25]. *Morbidity status* is self-reported: participants could indicate their morbidities on a 17-item list of conditions (no/yes), such as heart failure, joint damage and hearing disorders [24]. The number of morbidities were summed and the score ranges from 0 to 17. *Functional limitations* are measured with the modified Katz-15 instrument that assesses the ability to perform 15 activities of daily living (ADL) and instrumental activities of daily living (IADL) (yes/no) such as getting dressed, shopping and taking medication [27, 28]. The number of activities that respondents cannot do is summed, ranging from 0 to 15 with a higher score indicating more functional limitations.

*Frailty index* is calculated from 45 health deficits in the TOPICS-MDS [29], including the before mentioned self-reported health, cognitive functioning, social functioning, mental health, functional limitations and the five items of the EQ-5D [26]. The number of health problems of the older person is divided by the total number of 45 health problems; the score ranges from 0 to 1 with a higher score indicating a higher level of frailty [29, 30].

Demographic variables: gender, living arrangement (independent; in residential care or nursing home), marital status (married or cohabiting; widowed or single), ethnicity (native Dutch; first/second generation migrant), educational level (primary school or less; practical/secondary vocational training; some college/university degree) and age.

### Methods of analysis

The analyses were done in five steps combining quantitative and qualitative methods. First, we described the total sample, giving frequencies and percentages for the categorical variables and mean, standard deviations and range for the continuous variables (Table 1).

**Table 1:** Sample characteristics

	*N (43,704)*	*%*
Gender: Female	26,009	59.6
Living situation: Independently	38,321	89.6
Residential care setting or nursing home	4430	10.4
Marital status: Married/Cohabiting	21,368	49.8
Educational level Primary school or less	8639	22.7
Practical/secondary vocational training	22,913	60.2
Some college/university degree	6495	17.1
Ethnicity: Native Dutch	39,168	90.4
Self-reported health: Excellent	1533	3.8
Very good	3329	8.3
Good	17,150	42.7
Fair	15,379	38.3
Poor	2772	6.9
Self-reported health compared to 1 year ago: Much better	1030	2.6
Somewhat better	2488	6.2
About the same	21,639	54.1
Somewhat worse	11,487	28.7
Much worse	3370	8.4
Cognitive functioning:		
No problems with memory, attention & thinking	25,784	66.4
Some problems	12,187	31.4
Severe problems	856	2.2
Social functioning have problems with social activities: None of the time	18,804	46.4
A little of the time	7581	18.7
Some of the time	7668	18.9
Mostly	3414	8.4
All of the time	3043	7.5
	*Mean (SD)*	*Range*
Age	78.74 (7.12)	60.0–102.80
Mental health (0–100)^a^	73.69 (18.24)	0–100
Morbidity status (0–17 morbidities)^b^	2.89 (2.02)	0–17
Functional limitations (0–15 limitations)^c^	2.89 (3.30)	0–15
Frailty Index (0–1)^d^	0.23 (0.14)	0.00–0.85

^a^RAND Mental Health Subscale, higher scores represent better mental health;

^b^Self-reported number of morbidities, higher scores represent more morbidities;

^c^Modified Katz scale, higher scores represent more functional limitations;

^d^Frailty index, higher scores represent higher level of frailty

Second, we did latent class analysis (LCA) to identify subpopulations within a larger population of older people. LCA is a person-centred approach to identify unobserved groups of similar individuals (latent classes) based on observed variables. The aim of LCA is to find the best class solution; meaning the smallest number of latent classes describing the associations among a set of observed variables [31]. The observed variables we used in the LCA were self-reported health, social functioning and cognitive functioning as categorical variables and morbidity status, mental health, functional limitations as continuous variables. To avoid local likelihood maxima and inaccurate parameter estimates, we used 1000 multiple start values and 100 iterations [32]. For each class solution, we present the Akaike Information Criteria (AIC), Bayesian Information Criteria (BIC) and adjusted BIC (aBIC) which combine goodness of fit and parsimony [33]. We based the number of classes on the adjusted Lo-Mendell-Rubin likelihood test (bootstrap). The quality of the classification was determined by the entropy measure [34]. The various class solutions and model fit are presented in Additional file 1: Table S1. We used the Mplus 7.4 program. We based the final number of classes on the highest entropy score as it indicates the best quality classification.

Third, we described the final class solution according to the observed variables self-reported health, social functioning, cognitive functioning, morbidity status, mental health and functional limitations to identify the differences between them (see Table 2 and Additional file 2: Table S2).

**Table 2:**
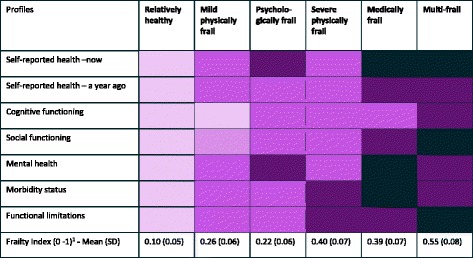
Six profiles of frail older people

NB: The darker the colour, the more severe the problems in the domain

^1^Frailty index, higher scores represent higher level of frailty

Fourth, we assessed the quality of the LCA classification with a focus group of older people (see Additional file 3 for the focus group protocol). In LCA, the value of the classes should also be interpreted qualitatively. The focus group participants were members of the Elderly Forum of the Geriatric Network Rotterdam, one of the eight regional networks in the NCEP. All 15 members of the Elderly Forum were invited to the focus group and eight (five males and three females) were able to attend. The profiles were presented textually for each of the final classes as identified by LCA: Older people in this profile experience their health as *[excellent/very good/good/fair/poor]* and state that their health is *[much better/somewhat better/about the same/somewhat worse/much worse]* compared to a year ago. They experience *[no/some/serious]* problems with their cognitive functioning. They experience problems with social activities *[a little/some/a good bit/most/all]* of the time. Their mean score on mental health is *[0–100].* They have *[0–17]* morbidities and need help with *[0–15]* daily activities. Besides the textual presentation, the final profiles were also presented together to provide a clear, visual overview. To validate the profiles, we asked the participants whether they recognized the profiles and if (how much) they could relate to them. In addition, we asked them to state which specific domain contributed the most to frailty in each of the profiles and invited them to rank the profiles from least to most frail. The focus group discussions were recorded and transcribed verbatim. We began the analysis by carefully rereading the transcript of the focus group several times and subsequently applied a data-driven approach to our thematic analysis per profile. We were looking for the interpretation of each of the profiles to understand the similarities and differences between the perceptions of the eight participants. Focus group quotes are presented with reference to respondents 1–8.

Fifth, we used the input of the focus group participants for additional (quantitative) analysis and further explored the class division quantitatively by looking into the distribution of demographic background variables (gender, living arrangement, marital status, ethnicity, educational level and age). We tested the relation between profiles and demographic variables and between the profiles and the frailty index with multinomial regression analysis (see Table 3). And we determined the scores of the frailty index, distribution of morbidities and functional limitations across the six subpopulations (see Tables 2, 4 and 5).

**Table 3:** Distribution (%) of demographic characteristics and the frailty index across the six frailty profiles

	Total (*n* = 43,704)	Relatively healthy (*n* = 17,580)	Mild physically frail (*n* = 6336)	Psychologically frail (*n* = 10,411)	Severe physically frail (*n* = 4522)	Medically frail (*n* = 3339)	Multi-frail (*n* = 1516)	Nagelkerke’s R square^a^
Gender: Female - %	59.6	50.6	68.9	59.8	70.3	71.4	63.9	0.03
Living situation: Independently - %	89.6	97.6	82.9	96.1	66.7	84.6	52.8	0.12
Marital status: Married/cohabiting - %	49.8	60.9	37.2	49.5	34.8	39.6	41.2	0.04
Educational level: - %								0.03
Primary school or less	22.7	15.8	27.4	21.7	32.0	31.1	34.0	
Practical/secondary vocational training	60.2	62.3	58.5	61.6	56.0	57.9	55.4	
Some college/university degree	17.1	21.9	14.1	16.7	11.9	11.1	10.7	
Ethnicity: Dutch native - %	90.4	91.2	90.6	89.4	90.6	88.7	89.4	0.00
Age: Mean (SD)	78.74 (7.13)	76.90 (6.19)	81.40 (7.14)	77.83 (6.61)	82.24 (7.97)	80.28 (6.99)	81.51 (9.24)	0.09

^a^explained variance of the multinomial regressions of the specific background characteristic on the division into six subpopulations

**Table 4:** Distribution (%) of morbidities across six frailty profiles

Morbidities - % of respondents indicating having a specific morbidity	Total (*n* = 43,704)	Relatively healthy (*n* = 17,580)	Mild physically frail (*n* = 6336)	Psychologically frail (*n* = 10,411)	Severe physically frail (*n* = 4522)	Medically frail (*n* = 3339)	Multi-frail (*n* = 1516)
Joint damage (osteoarthritis, rheumatoid wear) of hips or knees	44.1	29.8	45.4	53.4	49.5	70.2	48.9
Hearing problems	38.7	29.4	40.3	42.4	44.8	53.9	49.9
Vision disorders	32.0	18.5	35.0	35.7	42.3	56.5	46.8
Involuntary urinary loss	25.4	10.5	30.6	23.1	44.3	46.9	64.1
Diabetes	21.9	16.7	22.9	24.4	24.7	31.9	25.0
Heart failure	21.2	10.9	22.7	26.5	26.4	40.7	27.3
Osteoporosis	19.9	9.7	20.5	23.8	26.3	41.8	25.9
Asthma, chronic bronchitis, pulmonary emphysema or CARA/COPD	19.2	11.1	18.3	25.1	20.8	36.3	23.6
Dizziness with falling	16.1	6.2	15.1	18.9	22.4	41.0	28.9
A form of cancer (malignant disease)	11.2	7.6	10.8	14.1	12.7	18.5	11.6
Prostatism due to benign prostatic hyperplasia^a^	11.0	10.4	7.3	13.9	9.6	13.7	10.4
Stroke, brain haemorrhage, cerebral infarction or transient ischaemic attack	9.3	4.2	10.8	8.4	16.0	16.7	24.1
Depression	9.0	1.9	4.5	13.3	10.7	30.8	20.2
Fractures other than hip	6.7	3.3	8.0	6.1	11.8	11.9	12.8
Anxiety/panic disorder	6.0	1.3	2.6	8.2	7.0	21.8	14.8
Dementia	4.7	1.9	4.5	3.1	9.4	7.7	23.3
Hip fracture	3.8	1.3	5.0	2.8	9.2	5.8	10.2

^a^% of male respondents

**Table 5:** Distribution (%) of functional limitations based on a modified 15-item Katz Index across six frailty subpopulations

Functional limitations - % of respondents needing help with an activity	Total (*n* = 43,704)	Relatively healthy (*n* = 17,580)	Mild physically frail (*n* = 6336)	Psychologically frail (*n* = 10,411)	Severe physically frail (*n* = 4522)	Medically frail (*n* = 3339)	Multi-frail (*n* = 1516)
Do you need help taking care of your house?	54.6	19.1	91.4	49.3	98.4	93.6	99.3
Do you need help travelling?	36.2	5.2	67.9	17.7	91.7	75.1	99.0
Do you need help shopping?	32.3	3.3	61.7	14.9	91.0	70.6	98.9
Do you need help walking about?	29.9	4.4	55.9	13.3	81.0	57.0	88.1
Do you use incontinence products?	29.7	11.9	41.4	22.1	59.7	48.9	83.8
Do you need help preparing a meal?	24.7	3.9	38.7	6.8	81.0	41.7	98.3
Do you need help with taking a bath or shower?	21.8	1.0	32.1	3.5	84.4	35.4	99.4
Do you need help handling your finances?	19.2	7.8	28.6	6.0	49.6	23.2	83.3
Do you need help getting dressed?	15.4	0.6	18.2	1.7	67.2	20.6	97.2
Do you need help taking your medications?	12.6	1.0	14.6	2.8	42.8	14.7	83.4
Do you need help sitting down and getting up from a chair?	9.8	0.6	10.2	2.0	35.2	13.9	71.8
Do you need help toileting?	7.2	0.3	6.3	0.4	29.4	4.2	76.2
Do you need help using the telephone?	6.5	0.6	5.9	1.2	20.1	7.3	61.5
Do you need help brushing your hair or shaving?	5.3	0.1	1.9	0.1	17.2	2.4	71.4
Do you need help with eating?	2.6	0.1	1.0	0.1	6.0	1.6	43.6

The Results section presents the first and second steps of the analysis separately. The results of the third, fourth and fifth steps are combined and reported by profile.

## Results

Table 1 presents the sample characteristics. The total study population consisted of 43,704 older people, mostly female (59.6%) and with a mean (SD) age of 78.7 (7.1) years. Of the older people, 90% lived independently and half (49.8%) were married or cohabiting. The majority of the study population (60.2%) had a middle educational level (practical or secondary vocational training) and 90% was native Dutch. Health was perceived mostly as good or fair and 12% stated that their health was very good or excellent. Half of the population (54.1%) stated that their health was stable and a quarter indicated that it was somewhat worse compared to a year ago. Most of the study population (66.4%) had no problems with cognitive functioning, 27.8% had some problems and 2% had serious problems. Social activities were never hampered for 46%, whereas they were always hampered for 7.5%. On a scale from 0 to 100, the mean (SD) score on mental health was 73.7 (18.2). The older population had on average 2.9 morbidities (theoretical range: 0–17) and 2.9 functional limitations in terms of ADL and IADL (theoretical range: 0–15). The mean (SD) score on frailty index is 0.23 (0.14).

### Six profiles of frail older people

Latent class analysis with various class solutions identified six subpopulations within the population of older people. Additional file 1, Table S1  presents the model fit statistics of the various class solutions. In these different class solutions, two to three relatively big classes remained stable and the other classes became increasingly dispersed. In the eight-class solution, for example, two classes accounted for 50% of the study population and the remaining six classes were relatively small. We chose the six-class solution, based on the highest entropy score (0.81) which indicated the best quality classification.

Profile A (*‘relatively healthy’*) fundamentally differs from the other five profiles. Older people in this profile were relatively healthy; they indicated having good (mental) health and had very few problems across all the domains. They were not co-morbid; on average, they generally reported fewer than two morbidities and almost no functional limitations. When a functional limitation was reported, this was mostly related to household activities. The clear distinction between the *relatively healthy* and the other profiles is also demonstrated by differences in the background characteristics. The *relatively healthy* respondents are more likely to be male, younger, live independently and be married than the respondents in the other five profiles. Older people in the focus group clearly identified them as the least frail of the six profiles, “They are not frail compared to the rest, of course” (respondent 8). This is also confirmed by their mean score on the frailty index (0.10).

Profile B (*‘mild physically frail’*) features suffering from mild problems in the physical domain, and the focus group reinforced this characterization: “They need a lot of help. Needing help with four to five activities is quite a lot” (respondent 5). This profile reflected an initial loss of independence, particularly with regard to IADL activities. Almost all individuals required help in the household, and the vast majority needed help with travelling and shopping. Most still lived independently at home, but typically had no partner to help them with these activities. Moreover, *mild physically frail* people had multi-morbidity; joint damage and hearing problems were reported most frequently in this profile. Despite their functional limitations, their self-reported health and mental health were considerably good, underscoring the definition as “mild problems”. One focus group participant described the older people in this profile as follows: “The limitations are simply because of their age. But they’re not bothered by them and just go their own way” (respondent 4).

The types of problems in profile C (‘*psychologically frail’*) were rather different from the *mild physically frail* profile. Their reported health and mental health were relatively poor and social functioning was worse than in the *relative healthy, mild and severe physically frail* profiles. However, this profile reported only sporadic functional limitations; mostly related to problems in the household. Participants in the focus group still agreed that, despite their independence, the *psychologically frail* profile was rather frail. Their problems could partly be explained by their psychological condition, a relatively high percentage of people reported anxiety disorders and depression. However, the focus group also attributed the problems of this profile to their coping behaviour: “They treat every (minor) inconvenience as a major limitation or severe disease” (respondent 4). The participants of the focus group perceived the *psychologically frail* profile more frail than the *mild physically frail* profile. “People in this group are sensitive and will interpret things negatively which could lead to a self-fulfilling prophecy” (respondent 5). However, the mean score on the frailty index of this profile was lower than of the *mild physically frail* (0.22 respectively 0.26).

Profile D (‘*severe physically frail’*) was comparable to *mild physically frail* profile but here the physical problems were more severe and problems also expanded across other domains. On average, they had eight functional limitations, twice as many as found in the *mild physically frail* profile. Almost all people in the *severe physically frail* profile were hampered in IADL, such as taking care of the home, shopping and travelling. They needed considerably less help with the less physical IADL activities such as taking medication and handling finances. Older people in this profile also began encountering problems with ADL activities. For example, 80% said they needed help with showering. In this oldest profile, initial problems with cognitive and social functioning were prevalent. Despite their advanced age and severe limitations, they regarded their health as quite good. The mean score on the frailty index within was fairly high with 0.40. One focus group participant stated that the situation was delicate, “The moment anything goes wrong, they are in deep trouble but they’re not experiencing this yet” (respondent 7). The focus group agreed that people in the *severe physically frail* profile might be in denial of their frailty: “Their perception is positive even though the situation is serious” (respondent 5).

In the preceding four profiles, problems were mostly limited to one domain. However, in profile E (‘*medically frail*’) people accumulated problems in three domains –the physical, psychological and social – that seemed to be originated in their morbidities. People in this group mostly experienced fair or poor health in combination with a deterioration in their health compared to a year ago. Their social activities were frequently hampered by their physical condition and/or emotional problems. They experienced the worst mental health and the most morbidities of all profiles. These morbidities were psychological conditions such as depression and anxiety disorders but also physical conditions such as joint damage, dizziness with falling and heart failure. The score on the frailty index was similar to the *severe physically frail* profile. The older people in the focus group agreed that the older people in the *medically frail *profile were more frail. One of the focus group participants imagined that people with these kinds of morbidities “have physical problems that hamper them, for example in social activities, in particular compared to before” (respondent 4).

As for profile F (*‘multi-frail’*), in addition to problems in the physical, psychological and social domains, here people also had cognitive problems. They had the highest score on the frailty index (0.55) and also the focus group also characterized the *multi-frail* profile as the most frail, especially because of the cognitive problems combined with severe functional limitations. In the *multi-frail* profile, people had moderate to extreme cognitive problems and reported the highest percentage of dementia. On average, people in this profile needed help with 12 activities. The focus group described this state as “totally dependent” (respondent 1) and “needing help from morning to night” (respondent 8). They need help with almost all IADL and most ADL activities and almost half needed help with eating. Focus group participants felt that these older people should be institutionalized. Still, half of *multi-frail* profile lived independently, most often without a partner. Social functioning was hampered most frequently in this profile: “When you have these kinds of cognitive problems, I can image that you won’t undertake things yourself. I have 21 years of experience of dealing with my wife’s dementia. They don’t take the initiative, they become withdrawn” (respondent 6). The reported mental health in this profile was remarkable. As one respondent observed: “They still feel relatively good” (respondent 3). Another explained: “They’re not hampered by a sense of reality because of their cognitive problems” (respondent 5)*.*

## Discussion

Frailty is widely acknowledged to explain variations in health risks and is frequently used to select older people for care interventions. Yet, clearly, frailty is not binary but rather a heterogeneous identity. While the distinctions between the physical, psychological and social domain begin to distinguish the complexity of frailty, they do not fully capture the multifaceted concept of frailty. This research demonstrates that in fact six frailty profiles can be distinguished.

Our results show that ‘the’ frail older person does not exist. Frail older people are indeed a heterogeneous population, as is shown by our relatively high number of six profiles. Previous research on profiles on chronic conditions, general health status or physical frailty distinguished at most four profiles [14, 21–23, 35]. The *relatively healthy* profile remained a constant group in the different class solutions of our latent class analysis. Correspondingly, this relatively healthy group also emerged in previous research on subpopulations of older people [14, 22, 35] and it could be discussed whether the older people in this profile could be labelled as being frail. However, the remaining ‘relatively unhealthy’ (or frail) older people were divided into several smaller classes for which the six-class solution ultimately fitted the data best. The differences between the six profiles are substantial. Older people in the *relative healthy* profile have less than one functional limitation compared to the average of 12 functional limitations in the *multi-frail* profile. By including this full range of domains of functioning and the severity of the problems, our results enhance previous findings on frailty profiles. Our results showed that the physical domain is important [14, 22, 23] with two profiles of whom the severity of their problems clearly differed. Moreover, we found a separate profile for psychological frailty in contrast to other studies [35].

Our results showed specific patterns of underlying problems in different domains which confirm the complexity of frailty. The conventional distinction between the physical, psychological and social domains of frailty or determining the degree of frailty with frailty indexes barely do justice to this constellation of problems. Despite their comparable frailty index scores, older people in the *mild physically frail* and *psychologically frail* profiles experience rather contrasting problems. Also the *severe physically* and *medically* *frail* profiles had similar scores on the frailty index but the underlying problems clearly differed. In the *severe physically frail *profile the problems mostly originated in the physical domain whereas people in the *medically frail* profile suffer from a combination of problems in the physical, psychological and social domains. In the *multi-frail* profile the constellation also extended to the cognitive domain of functioning. Unlike problems in the physical and psychological domains, problems in the social domain did not emerge in a separate subpopulation. Social frailty seems related to problems in the other domains, for example to morbidities or functional limitations but the direction of the relation between health and social functioning still remains unclear [36]. Our study provides valuable insights in the complex interaction of problems of frail older people.

Underlying problems in the different domains may not contribute equally to the degree of frailty. Focus group participants carefully weighed the problems in all domains and were well able to rank the six profiles from least to most frail. This ranking did not correspond with the scores on the frailty index. For the focus group, frailty was synonymous with losing independence and respondents clearly perceived *multi-frail *profile as the most frail because of the cognitive problems and functional limitations which made people in this group extremely dependent. While considering the frailty profile rankings, the focus group weighed off the assets and deficits. Not all domains were deficits according to them; they could also be assets that help people cope with their problems. The (mis)balance between assets and deficits resulted in frailty (see also [3, 37]). The focus group clearly mentioned this in relation to the difference between the *mild physically frail* and *psychologically frail *profiles. Although the *mild physically frail* profile had four times more functional limitations than the *psychologically frail*, the latter was still perceived as more frail because people in this group had a limited capacity to cope with ageing and deterioration of their health.

Finally, our study challenges the relevance of demographic variables in the conceptualization of frailty. Age is too restricted a factor to predict health status, as previous research has confirmed [4, 5, 7]. Also, the relation of frailty to other demographic variables such as gender, marital status, ethnicity and educational level is limited. Only living arrangement related moderately to the frailty subpopulations but it could be considered an outcome of frailty rather than an antecedent.

### Strengths and limitations

The main strength of this study is its strong empirical base for frailty profiles. We were able to use data from TOPICS-MDS, a large data-sharing initiative that contains data on older people from all around the Netherlands. The large sample, combined with considerations of several domains of functioning makes the current research valuable. The quantitative results were complemented with a qualitative approach, which also adds value. The focus group enabled us to further validate the profiles and to understand the older person’s perspective on frailty.

The first limitation is that even though the 54 TOPICS-MDS projects generally focused on older people at risk or frail older people, their sampling frame and inclusion criteria substantially differed. Older people were included based on functional limitations or were screened frailty instruments questionnaires such as Groningen Frailty Indicator, whereas other projects adopted an age criterion. Our study included all 54 projects and our only selection criterion was age; people 60 years and older were included. As the focus group also indicated, there is still disagreement on what is regarded as ‘old’ and 60 years might be relatively young. The literature recommends including people of 70 years and older for frailty interventions [38] but a systematic search of the literature revealed that different age criteria are adopted [18]. We decided to include everyone 60 years and older in order to also include older people with a migrant background in whom ageing begins at a younger age and who often experience worse physical and emotional health than people born in the Netherlands [39]. We expect that the relatively young sample may not have influenced our results since the effect of age on the frailty profiles was negligible. Including all people of 60 years and older might have also resulted in the rather large group of older people in the *relatively healthy* profile and it remains unclear whether these older people could actually be considered as being frail. They were not perceived as frail according to the participants of the focus group and their score on the frailty index was also below the general cut-off point of 0.20 [30]. Nevertheless, some older people in this *relatively healthy* profile were identified as being frail by the Groningen Frailty Indicator.

The second limitation was the formulation of the social functioning item that might possible have affected our results. The item was phrased as: “During the past four weeks, to what extent has your physical health or emotional problems interfered with your normal social activities with family, friends, neighbours, or groups (like visiting friends or close relatives)?” This phrasing related social functioning directly to both physical and psychological functioning and might have contributed to the absence of a separate social frailty profile.

### Recommendations

The most important implication of our study is that we should take the heterogeneity of frailty into consideration in research, policy and practice. Future research should endeavour to further validate our six profiles. The focus group with older people was a good starting point but the profiles could also be validated by professionals and policy makers. Our identification effort should also be replicated on other databases of frail older people and with other measurement instruments, for example for social functioning. Testing the validity of these profiles would also involve examining frailty trajectories. In other words, our cross-sectional latent class analysis could be complemented with a latent transition analysis [34] which could account for the dynamic and progressive character of frailty [9]. In this regard, it would be beneficial to explore whether *mild physically frail* profile eventually transfer to the *severe physically frail* profile or how the trajectory of relatively healthy people progresses.

Our research also has implications for selecting the appropriate target groups for care interventions. The psycho-social domains of frailty are deemed important and focusing on the physical domain of frailty and functional limitations by professionals, researchers and policy makers could be too restricted. Our research showed that a relatively large group suffers from problems in the psychological domain without having problems in the physical domains in terms of functional limitations. It is important to target this profile for care interventions. Instruments with a broad perspective including the psycho-social domains such as frailty indexes [16, 30] and the Groningen Frailty Indicator [40] sum the total numbers of health problems which implies that they do not differentiate between the types of underlying problems or weigh the different domains. Older people with the same score on the frailty index or Groningen Frailty Indicator could have different underlying problems and frailty profiles [17]. The *severe physically frail* had a similar frailty index as the *medically frail* but their psychological and social functioning was not hampered. The *medically frail* profile showed that their problems related to morbidities extended to severe problems in the psychological and social domains. These problems should be also be acknowledged by health care professionals who are originally trained to have a disease-specific approach (Lette et al., 2015). In other words, the balance between deficits *and* assets in relation to frailty should be further explored in practice, policy and research. Assets could be coping style, resilience [41] or resources such as older people’s social network [3], which should be considered in the conceptualization and measurement of frailty.

Lastly, the six frailty profiles could be used to develop tailor-made care interventions for each profile rather than producing one-size-fits-all care. The heterogeneity of frailty should be incorporated in the evaluation of these interventions. Currently, traditional evaluation research is not acknowledging this heterogeneity in, for example, integrated care, which is misaligned with its goal to provide person-centred care with a holistic view of the individual [42]. So far, the effects of integrated care on health outcomes is limited [18]. Concurrently, the more heterogeneous a population is, the harder it is to achieve effectiveness [38, 43]. A possible explanation for the limited effectiveness of integrated care could be that the care professionals involved – and particularly researchers conducting the evaluation research – generally perceive frail older people as a homogeneous group. Evaluation research on integrated care could be replicated by incorporating the frailty profiles to gain deeper insight into the effectiveness of integrated care interventions. It would be beneficial to explore whether integrated is (more) effective on specific outcomes for each of the six profiles separately. Future research should explore, for example, whether integrated care is more effective in terms of mental health for the psychological frail than for the mild physical profile.

## Conclusions

Frail older people are a heterogeneous population and ‘the’ frail older person does not exist. Six frailty profiles were developed on the full range of domains of functioning and the severity of these domains. Specific patterns of underlying problems in different domains emerged. Acknowledging the heterogeneity by frailty profiles is crucial for tailoring and evaluating interventions and developing policy for frail older people.

## Additional files

Additional file 1: **Table S1.** Model fit – latent class analysis. (DOCX 13 kb)

Additional file 2: **Table S2.** Conditional probabilities per profile. (DOCX 15 kb)

Additional file 3: Focus group protocol. (DOCX 20 kb)

## Abbreviations

aBIC: Adjusted Bayesian Information Criteria
ADL: Activities of daily living
AIC: Akaike Information Criteria
BIC: Bayesian Information Criteria
IADL: Instrumental activities of daily living
LCA: Latent class analysis
SD: Standard Deviation
TOPICS-MDS: The Older Persons and Informal Caregivers Survey Minimum Dataset

## References

[1] United Nations, Department of Economic and Social Affairs, Populations Division: World Population Ageing 2015. 2015, (ST/ESA/SER.A/390):.

[2] World Health Organization: World report on ageing and health. Geneva: WHO. ISBN: 978 924 156504 2.

[3] Rockwood K, Fox RA, Stolee P, Robertson D, Beattie L (1994). Frailty in elderly people: an evolving concept. CMAJ.

[4] Slaets JPJ (2006). Vulnerability in the elderly: frailty. Med Clin North Am.

[5] Schuurmans H, Steverink N, Lindenberg S, Frieswijk N, Slaets JP (2004). Old or frail: what tells us more?. J Gerontol Ser A Biol Med Sci.

[6] Van Kempen JAL, Schers HJ, Jacobs A, Zuidema SU, Ruikes F, Robben SHM, RJF M, Olde Rikkert MGM (2013). Development of an instrument for the identification of frail older people as a target population for integrated care. Br J Gen Pract.

[7] Lacas A, Rockwood K (2012). Frailty in primary care: a review of its conceptualization and implications for practice. BMC Med.

[8] Fried LP, Tangen CM, Walston J, Newman AB, Hirsch C, Gottdiener J, Seeman T, Tracy R, Kop WJ, Burke G, McBurnie MA, Cardiovascular Health Study Collaborative Research Group (2001). Frailty in older adults: evidence for a phenotype. J Gerontol A Biol Sci Med Sci.

[9] Gobbens R, Luijkx K, Wijnen-Sponselee MT, Schols J (2010). Towards an integral conceptual model of frailty. J Nutr Health Aging.

[10] Clegg A, Young J, Iliffe S, Rikkert MO, Rockwood K (2013). Frailty in elderly people. Lancet.

[11] Dent E, Kowal P, Hoogendijk EO (2016). Frailty measurement in research and clinical practice: a review. Eur J Intern Med.

[12] Manthorpe J, Iliffe S (2015). Frailty – from bedside to buzzword?. J Integrated Care.

[13] Markle-Reid M, Browne G (2003). Conceptualizations of frailty in relation to older adults. J Adv Nurs.

[14] Lafortune L, Béland F, Bergman H, Ankri J. Health status transitions in community-living elderly with complex care needs: a latent class approach. BMC Geriatr. 2009;9(6). 10.1186/1471-2318-9-6. 10.1186/1471-2318-9-6 PMC2645408 19192295

[15] Rockwood K, Mitnitski A (2007). Frailty in relation to the accumulation of deficits. J Gerontol Ser A Biol Med Sci.

[16] Rockwood K, Song X, MacKnight C, Bergman H, Hogan DB, McDowell I, Mitnitski A (2005). A global clinical measure of fitness and frailty in elderly people. CMAJ.

[17] Metzelthin SF, Daniëls R, van Rossum E, de Witte L, van den Heuvel WJA, Kempen GI (2010). The psychometric properties of three self-report screening instruments for identifying frail older people in the community. BMC Public Health.

[18] Eklund K, Wilhelmson K. Outcomes of coordinated and integrated interventions targeting frail elderly people: a systematic review of randomized controlled trails. Health Soc Care Community. 2009;17(5):447–58 10.1111/j.1365-2524.2009.00844.x 19245421

[19] Newcomer SR, Steiner JF, Bayliss EA (2011). Identifying subgroups of complex patients with cluster analysis. Am J Manag Care.

[20] Olaya B, Moneta MV, Caballero FF, Tyrovolas S, Bayes I, Ayuso-Mateos JL, Haro JM (2017). Latent class analysis of multimorbidity patterns and associated outcomes in Spanish older adults: a prospective cohort study. BMC Geriatr.

[21] Gellert P, von Berenberg P, Zahn T, Neuwirth J, Kuhlmey A, Dräger D. Multimorbidity profiles in German centenarians: a latent class analysis of health insurance data. J Aging Health. 2017. p. 898264317737894. 10.1177/0898264317737894. [Epub ahead of print] 10.1177/0898264317737894 29254430

[22] Liu LF, Tian WH, Yao HP (2014). The heterogeneous health latent classes of elderly people and their socio-demographic characteristics in Taiwan. Arch Gerontol Geriatr.

[23] Liu L, Guo C, Lee W, Chen L, Hwang A, Lin M, Peng L, Chen L, Liang K (2017). Subtypes of physical frailty: latent class analysis and associations with clinical characteristics and outcomes. Sci Rep.

[24] Lutomski JE, Baars MAE, Schalk BWM, Boter H, Buurman BM, den Elzen WPJ, Jansen APD, Kempen G, Steunenberg B, Steyerberg EW, Rikkert M, Melis RJF (2013). The development of the older persons and informal caregivers survey minimum DataSet (TOPICS-MDS): a large-scale data sharing initiative. PLoS One.

[25] Van der Zee K, Sanderman R: RAND-36. Groningen: Northern Centre for Health Care Research, University of Groningen, the Netherlands 1993, 28.

[26] Krabbe PF, Stouthard ME, Essink-Bot M, Bonsel GJ (1999). The effect of adding a cognitive dimension to the EuroQol multiattribute health-status classification system. J Clin Epidemiol.

[27] Laan W, Zuithoff A, Drubbel I, Bleijenberg N, Numans M, De Wit N, Schuurmans M (2014). Validity and reliability of the Katz-15 scale to measure unfavorable health outcomes in community-dwelling older people. J Nutr Health Aging.

[28] Weinberger M, Samsa GP, Schmader K, Greenberg SM, Carr DB, Wildman DS (1992). Comparing proxy and patients’ perceptions of patients’ functional status: results from an outpatient geriatric clinic. J Am Geriatr Soc.

[29] Lutomski JE, Baars MA, Kempen JA, Buurman BM, Elzen WP, Jansen AP, Kempen GI, Krabbe PF, Steunenberg B, Steyerberg EW (2013). Validation of a frailty index from the older persons and informal caregivers survey minimum data set. J Am Geriatr Soc.

[30] Searle SD, Mitnitski A, Gahbauer EA, Gill TM, Rockwood K (2008). A standard procedure for creating a frailty index. BMC Geriatr.

[31] Muthén B, Muthén LK (2000). Integrating person-centered and variable-centered analyses: growth mixture modeling with latent trajectory classes. Alcohol Clin Exp Res.

[32] Geiser C (2012). Data analysis with Mplus*:* Guilford press.

[33] Hagenaars JA, McCutcheon AL (2002). Applied latent class analysis.

[34] Muthen LK, Muthen BO (1998). Mplus [computer software].

[35] Ng CW, Luo N, Heng BH (2014). Health status profiles in community-dwelling elderly using self-reported health indicators: a latent class analysis. Qual Life Res.

[36] George LK, Binstock RH, George LK, Marshall VW, Myers GC, Schulz JH (1996). Social factors and illness. Handbook of aging and the social sciences.

[37] Van Campen C: Frail older persons in the Netherlands, Den Haag: SCP 2011,32.

[38] Ferrucci L, Guralnik JM, Studenski S, Fried LP, Cutler GB, Walston JD (2004). Designing randomized, controlled trials aimed at preventing or delaying functional decline and disability in frail, older persons: a consensus report. J Am Geriatr Soc.

[39] Den Draak M, De Klerk M: Elderly migrants. Den Haag, SCP, 2011, 35.

[40] Peters LL, Boter H, Slaets JPJ, Buskens E (2013). Development and measurement properties of the self assessment version of the INTERMED for the elderly to assess case complexity. JPsychosomatRes.

[41] Wiles JL, Wild K, Kerse N, Allen RE (2012). Resilience from the point of view of older people:‘There's still life beyond a funny knee. Soc Sci Med.

[42] Mur-Veeman I, Hardy B, Steenbergen M, Wistow G (2003). Development of integrated care in England and the Netherlands: managing across public–private boundaries. Health Policy.

[43] Lette M, Baan CA, van den Berg M, de Bruin SR. Initiatives on early detection and intervention to proactively identify health and social problems in older people: experiences from the Netherlands. BMC Geriatr. 2015;15(143). 10.1186/s12877-015-0131-z. 10.1186/s12877-015-0131-z PMC4628317 26518369

