# Supplementary material for: The frail older person does not exist: development of frailty profiles with latent class analysis
Source: BMC Geriatr. 2018 Apr 4;18:84. doi: 10.1186/s12877-018-0776-5 (PMC5885355; doi:10.1186/s12877-018-0776-5)
Supplement: Supplementary file 1 — Table S1. Model fit – latent class analysis. (DOCX 13 kb) [file 12877_2018_776_MOESM1_ESM.docx]

**Additional file 1: Table S1: Model fit – latent class analysis**

|  | 2 classes | 3 classes | 4 classes | 5 classes | 6 classes | 7 classes | 8 classes | 9 classes | 10 classes |
| --- | --- | --- | --- | --- | --- | --- | --- | --- | --- |
| Information criterion |  |  |  |  |  |  |  |  |  |
| AIC | 1057575.878 | 1044960.251 | 1038571.226 | 1031816.004 | 1028366.429 | 1025009.292 | 1022780.354 | 1020685.911 | 1019009.583 |
| BIC | 1057905.916 | 1045446.622 | 1039213.931 | 1032615.042 | 1029321.801 | 1026120.997 | 1024048.392 | 1022110.283 | 1020590.288 |
| Adjusted BIC | 1057785.151 | 1045268.653 | 1038978.758 | 1032322.665 | 1028972.220 | 1025714.212 | 1023584.403 | 1021589.089 | 1020011.891 |
|  |  |  |  |  |  |  |  |  |  |
| LMT LRT |  |  |  |  |  |  |  |  |  |
| Log likelihood value | -550688.022 | -528749.939 | -522424.125 | -519211.613 | -515816.002 | -514073.215 | -512376.646 | -511244.177 | -510178.955 |
| -2 difference in log likelihood | 43876.165 | 12651.627 | 6425.025 | 6791.223 | 3485.574 | 3393.137 | 2264.939 | 2130.443 | 1712.328 |
| p value | <0.0001 | <0.0001 | <0.0001 | <0.0001 | 0.0206 | 0.0007 | <0.0001 | <0.00001 | <0.00001 |
|  |  |  |  |  |  |  |  |  |  |
| Adjusted LMR LRT | 43649.220 | 12586.188 | 6391.792 | 6756.096 | 3467.546 | 3375.586 | 2253.224 | 2119.423 | 1703.471 |
| p value | <0.0001 | <0.0001 | <0.0001 | <0.0001 | 0.0213 | 0.0007 | <0.0001 | <0.0001 | <0.0001 |
|  |  |  |  |  |  |  |  |  |  |
| Entropy | 0.737 | 0.766 | 0.770 | 0.789 | 0.810 | 0.797 | 0.776 | 0.782 | 0.783 |
| Condition no. | 0.513E-04 | 0.280E-04 | 0.168E-03 | 0.137E-03 | 0.140E-03 | 0.497E-04 | 0.125E-03 | 0.111E-03 | 0.583E-04 |
