# Supplementary material for: The frail older person does not exist: development of frailty profiles with latent class analysis
Source: BMC Geriatr. 2018 Apr 4;18:84. doi: 10.1186/s12877-018-0776-5 (PMC5885355; doi:10.1186/s12877-018-0776-5)
Supplement: Supplementary file 2 — Table S2. Conditional probabilities per profile. (DOCX 15 kb) [file 12877_2018_776_MOESM2_ESM.docx]

**Additional file 2: Table S2: Conditional probabilities per profile**

|  | *Total* | A  Relatively healthy | B  Mild physically frail | C  Psycholo-gically frail | D  Severe physically frail | E  Medically frail | F  Multi-frail |
| --- | --- | --- | --- | --- | --- | --- | --- |
| **Self-reported health:** 1 excellent | *3.8* | 8.1 | 2.6 | 0.2 | 1.9 | 0.1 | 1.4 |
| 2 very good | *8.3* | 17.4 | 5.2 | 1.0 | 3.5 | 0.4 | 2.1 |
| 3 good | *42.7* | 61.8 | 47.2 | 28.1 | 34.6 | 4.7 | 21.9 |
| 4 fair | *38.3* | 12.6 | 40.9 | 64.8 | 47.6 | 62.6 | 45.5 |
| 5 poor | *6.9* | 0.1 | 4.1 | 5.9 | 12.3 | 32.2 | 29.1 |
| **Self-reported health:** compared to year ago:  1 much better | *2.6* | 3.8 | 2.8 | 1.6 | 1.8 | 0.3 | 1.7 |
| 2 somewhat better | *6.2* | 5.9 | 7.4 | 6.6 | 6.8 | 4.1 | 5.4 |
| 3 about the same | *54.1* | 80.8 | 48.5 | 39.5 | 35.5 | 14.4 | 24.5 |
| 4 somewhat worse | *28.7* | 9.1 | 34.6 | 44.4 | 39.3 | 48.2 | 36.8 |
| 5 much worse | *8.4* | 0.4 | 6.8 | 7.8 | 16.5 | 33.0 | 31.6 |
| **Cognitive functioning:**  1 no problems | *66.4* | 82.0 | 66.5 | 57.7 | 55.2 | 45.0 | 36.2 |
| 2 some | *31.4* | 17.8 | 31.8 | 41.1 | 39.6 | 49.6 | 45.1 |
| 3 severe | *2.2* | 0.1 | 1.7 | 1.2 | 5.2 | 5.5 | 18.7 |
| **Social functioning**: problems with social activities:  1 none of the time | *46.4* | 76.3 | 44.8 | 21.4 | 27.2 | 8.7 | 13.8 |
| 2 a little of the time | *18.7* | 17.7 | 19.0 | 25.8 | 14.8 | 9.3 | 9.4 |
| 3 some of the time | *18.9* | 5.1 | 21.1 | 36.2 | 23.3 | 28.4 | 15.6 |
| 4 most of the time | *8.4* | 0.5 | 8.7 | 10.9 | 15.9 | 27.3 | 21.6 |
| 5 all of the time | *7.5* | 0.5 | 6.5 | 5.6 | 18.8 | 26.4 | 39.6 |
| **Mental health:** (0–100) mean (SD) | *73.69* | 83.75 (0.22) | 78.21 (0.54) | 64.86 (1.19) | 70.21 (2.27) | 50.60 (1.65) | 59.75 (1.73) |
| **Morbidity status:** (0–17 diseases) mean (SD) | *2.88* | 1.68 (0.03) | 2.97 (0.07) | 3.18 (0.08) | 3.77 (0.20) | 5.15 (0.16) | 4.45 (0.14) |
| **Functional limitations:** (0–15 limitations) mean (SD) | *2.89* | 0.56 (0.02) | 4.58 (0.10) | 1.27 (0.06) | 8.33 (0.07) | 4.88 (0.41) | 12.25 (0.16) |
| % | *100* | 40 | 14 | 24 | 10 | 8 | 3 |
| N | 43,704 | 17,580 | 6,336 | 10,411 | 4,522 | 3,339 | 1,516 |

^1^RAND Mental Health Subscale, higher scores represent better mental health; ^2^Self-reported number of morbidities, higher scores represent

more morbidities; ^3^Modified Katz scale, higher scores represent more functional limitations.
