# Supplementary material for: The frail older person does not exist: development of frailty profiles with latent class analysis
Source: BMC Geriatr. 2018 Apr 4;18:84. doi: 10.1186/s12877-018-0776-5 (PMC5885355; doi:10.1186/s12877-018-0776-5)
Supplement: Supplementary file 3 — Focus group protocol. (DOCX 20 kb) [file 12877_2018_776_MOESM3_ESM.docx]

**Additional file 3 Focus group protocol**

**Before the focus group**

All participants of the focus group get a paper form with 4 general questions. The participants were asked to fill in this form before the focus group had started.

1. How do you define frailty?
2. When would you consider an older person as being frail?
3. What do you think of the idea to divide frail older people in different profiles?
4. In what profiles would you personally divide the population of frail older people?

**During the focus group**

The main researcher of the project (WL) introduced herself and gave a presentation on the research project and the aim of the focus group.

**Objective of the project:**

Age explains health differences between older people insufficiently

Therefore: frailty

But: also big differences between frail older people

**Research project:**

TOPICS-MDS data of more than 40,000 older people

Looking for profiles of frail older people…

… to include in future research on integrated care

**Discuss the answer to 4 questions on the paper form:**

1. How do you define frailty?
2. When would you consider an older person as being frail?
3. What do you think of the idea to divide frail older people in different profiles?
4. In what profiles would you personally divide the population of frail older people?

**Explanation of the profiles:**

On what features do we characterize the profiles?

Perceived health

- Now
- Compared to one year ago

Problems with memory

- Problems with memory, attention and thinking

Problems with social activities

- Hampered with social activities by physical health or emotional problems

Mental health

- How often do you feel nervous or down

Diseases

- List of 17 diseases, for example diabetes or hearing problems

Needing help with daily activities

- Number of activities that people need help with, for example dressing or household activities

**6 profiles**

The data-analysis shows:

- 40,000 older people could be divided into 6 profiles

This means:

Older people within each profile have more in common with each other than with older people from the other profiles.

**Important aims of today**

Interpreting the profiles

Questions:

- Do you recognize the profiles?
- Do you know examples from your own environment of older people within the profiles?
- What terminology would you use to describe the profiles?
- What specific domain contributed most to frailty in each profile?
- Could you rank the profiles from least to most frail?
- By what profiles would you recommend the following interventions:
  - Exercise programme
  - Social activities
  - Early detection
  - Case management
  - Integrated care model

The six profiles were presented in text and in one table to provide a clear overview:

**Profile 1**

Older people in profile 1 report **good health** and state that their health is **about the same** compared to a year ago.

They experience **no** problems with cognitive functioning. They have problems with social activities **none of the time**.

Their mean score on mental health is **83.**

They have **1.7** morbidities and need help with **0.6** activities.

**Profile 2**

Older people in profile 2 report **good to fair health** and state that their health is **about the same to somewhat worse** compared to a year ago.

They experience **no** problems with cognitive functioning.

They have problems with social activities **none to little of the time**.

Their mean score on mental health is **78**.

They have **3** morbidities and need help with **4.6** activities.

**Profile 3**

Older people in profile 3 report **fair health** and state that their health is **somewhat worse to about the same** compared to a year ago.

They experience **no to some** problems with cognitive functioning.

They have problems with social activities **little to some of the time**.

Their mean score on mental health is **65**.

They have **3.2** morbidities and need help with **1.3** activities.

**Profile 4**

Older people in profile 4 report **fair to good health** and state that their health is **somewhat worse to about the same** compared to a year ago.

They experience **no to some** problems with cognitive functioning.

They have problems with social activities **none, little to some of the time**.

Their mean score on mental health is **70**.

They have **3.8** morbidities and need help with **8.3** activities.

**Profile 5**^[[1]](#footnote-1)^

Older people in profile 5 report **fair to poor health** and state that their health is **somewhat to much worse** compared to a year ago.

They experience **some to severe** problems with cognitive functioning.

They have problems with social activities **most to all the time**.

Their mean score on mental health is **60**.

They have **4.5** morbidities and need help with **12.2** activities.

**Profile 6**^1^

Older people in profile 6 report **fair to poor health** and state that their health is **somewhat to much worse** compared to a year ago.

They experience **no to some** problems with cognitive functioning.

They have problems with social activities **some, most to all the time**.

Their mean score on mental health is **51**.

They have **5.2** morbidities and need help with **4.9** activities.

**Six profiles of frail older people**

| Profile | 1 | 2 | 3 | 4 | 5^1^ | 6^1^ |
| --- | --- | --- | --- | --- | --- | --- |
| Perceived health – now |  |  |  |  |  |  |
| Perceived health – a year ago |  |  |  |  |  |  |
| Problems with memory |  |  |  |  |  |  |
| Problems with social activities |  |  |  |  |  |  |
| Mental health |  |  |  |  |  |  |
| Diseases |  |  |  |  |  |  |
| Needing help with daily activities |  |  |  |  |  |  |

1. In the focus group the order of the profiles was different than in the article. The participants of the focus group strongly agreed that profile 5 was more more frail than profile 6. Therefore, we changed the order in the article: profile 5 was changed into profile F (multi-frail) and profile 6 was changed into profile E (medically frail). [↑](#footnote-ref-1)
